# 

**Published:** 1900-05

**Authors:** 


### South Texas Medical Association.

HOUSTON, TEXAS, April 21, 1900.

DEAR DOCTOR: Our next meeting will be held in Beaumont on the 23rd and 24th of May. We trust that you will not only be in attendance, but that you will also honor the Association with a paper.

The program will be printed May 10th, therefore, it will be necessary to have the title of your paper on or before the 8th of May.

Fraternally yours,

J. H. REUSS, President, Cuero.

D. S. WIER, Secretary, Houston.

